# Retraction: Responses of Rapid Viscoanalyzer Profile and Other Rice Grain Qualities to Exogenously Applied Plant Growth Regulators under High Day and High Night Temperatures

**DOI:** 10.1371/journal.pone.0310933

**Published:** 2024-09-18

**Authors:** The PLOS ONE Editors

This article [1] was investigated by PLOS due to indicators that suggested it may be linked to a series of submissions with related integrity concerns.

For this article [1], we noted concerns about the article’s authorship and compliance with the PLOS Data Availability policy. Contrary to the Data Availability statement, the individual-level data underlying the published results were not provided within the paper.

The underlying data were provided by the first author upon editorial request. PLOS and an independent member of the *PLOS ONE* Editorial Board rereviewed the article and the underlying data provided. During this review, several additional concerns were identified, including errors and discrepancies within the underlying dataset, and between the underlying data and the published results. In addition, the Editorial Board member raised concerns about the study design and about results statements that were not supported by the data.

The issues were not resolved in follow-up discussions.

In light of the above concerns that question the reliability of the published results, the *PLOS ONE* Editors retract this article.

SF did not agree with the retraction and stands by the article’s findings. SHussain, SS, SHassan, BSC, FK, MZI, AU, CW, AAB, HA, A, WN, BS, MT, and JH either did not respond directly or could not be reached.
